# Comparative Study of Methods for Caries Risk Evaluation: CAMBRA, the Cariogram, and Caries Risk Semaphore

**DOI:** 10.3390/jcm14155378

**Published:** 2025-07-30

**Authors:** Iris Català-Benavent, José Enrique Iranzo-Cortés, Teresa Almerich-Torres, Cecilia Fabiana Márquez-Arrico, José Manuel Almerich-Silla, José María Montiel-Company

**Affiliations:** Facultat de Medicina i Odontologia, Department of Stomatology, Universitat de València, C/Gascó Oliag 1, 46010 Valencia, Spain; icabe3@alumni.uv.es (I.C.-B.); teresa.almerich@uv.es (T.A.-T.); cecilia.marquez@uv.es (C.F.M.-A.); jose.m.almerich@uv.es (J.M.A.-S.); jose.maria.montiel@uv.es (J.M.M.-C.)

**Keywords:** dental caries, risk, prevention, Cariogram, CAMBRA

## Abstract

**Background/Objectives:** Caries risk assessment is essential for the management of dental caries. There are different assessment methods with the most commonly used being CAMBRA, the Cariogram, and Caries Risk Semaphore (CRS). The aim of this study was to determine the diagnostic agreement between the three different caries risk assessment methods mentioned above. **Methods:** This study was conducted in the Dental Clinic of the University of Valencia by Preventive and Community Dentistry II students on patients examined during clinical practices (n = 672). Patients were evaluated to determine their caries risk using the three methods named above. A descriptive analysis of the sample was performed, and diagnostic agreement was assessed using the Kappa coefficient. **Results:** According to CRS, 321 patients (48%) showed high risk, 96 patients (14%) moderate risk, and 255 (38%) low risk. The highest diagnostic agreement was found between CRS and CAMBRA, with an unweighted Kappa of 0.36. Regarding risk severity assessments, the highest Kappa was also observed between CRS and CAMBRA, with a Kappa of 0.46 for low risk, 0.14 for moderate risk, and 0.40 for high risk. **Conclusions:** There is an important heterogeneity in the obtained results. This highlights the need to further study different caries risk assessment methods and determine their predictive capacity to choose the one that yields the best outcome.

## 1. Introduction

Dental caries can be defined as a localized pathological process of external origin that begins after tooth eruption and leads to demineralization of the hard tissue, eventually resulting in cavity formation [1]. If left untreated, dental caries can negatively impact health, child development, and family well-being [2]. Additionally, children with caries in their primary dentition are three times more likely to experience carious lesions in their permanent dentition [3]. Dental caries remains a significant health issue for both adults and children [1,4,5,6].

This multifactorial disease involves various bacteria, among which we highlight those that present acidophilic metabolism. Disruption of the dental biofilm (plaque) due to dietary habits, salivary alterations, inappropriate preventive strategies, or poor oral hygiene can lead to caries lesions [1,6]. Given its multifactorial nature and the influence of social determinants of health, preventive and therapeutic measures must be tailored to individual patients rather than generalized approaches. Since it is a multifactorial disease, it is important to consider that the interaction between acidogenic bacteria, a diet rich in sugars, and oral hygiene will determine the appearance of dental caries, among other aggravating factors [3].

In epidemiology, risk refers to the probability of a specific outcome occurring after exposure to a defined factor. Effective caries management relies on assessing an individual’s caries risk and developing personalized treatment plans based on documented information [7]. Regular evaluation of caries risk is crucial to monitor changes in oral health over time [7]. Currently, diagnostic methods such as caries management by risk assessment (CAMBRA) and the Cariogram are used to accurately assess caries risk [8,9].

CAMBRA, introduced by the California Dental Association (CDA) in 2002, aims to identify factors contributing to caries development and implement corrective measures [6,8,10,11,12,13,14,15]. This approach evaluates caries risk based on risk factors, protective factors, and disease indicators. CAMBRA considers caries as a continuous balance or imbalance between pathological and protective factors [2]. Dentists can determine behaviors that increase a patient’s caries risk and progression [6,8,10,11,12,13,14,15]. CAMBRA includes versions for children aged 0–5 years and those older than 6 years. The questionnaire must be completed by the dentist after the dental exam and an interview with the child and his or her parents and is adapted for each age group, and it covers disease indicators, risk factors, and protective factors. Affirmative risk factors are worth one point, positive disease indicators are worth two points, and each protective factor is worth one point less. Patients are classified as low risk (−9 to 4 points) or high risk (5 to 19 points) based on the results. A high-risk patient is deemed to be at extreme risk if they also have a significant decrease in salivary flow [16].

The Cariogram has been developed over the years, primarily by personnel at the University of Malmö in Sweden, including Bratthall and Petersson [17,18,19,20]. It is a computer application that assesses etiological factors related to caries, graphically displays a patient’s potential risk, and suggests preventive and treatment strategies. The Cariogram evaluates various risk factors, calculates the overall risk of developing a new severe lesion, and presents the results on a 0–100% graph [17,18,19,20].

Using an embedded formula, the program generates a circular chart depicting common caries risk scenarios in five color-coded areas: dark blue represents ‘diet’; red corresponds to ‘bacteria’; light blue indicates ‘susceptibility’; yellow represents ‘circumstances’; and green estimates the probability of avoiding new caries. Based on this last sector, Cariogram results can be classified as follows: low risk (61% or higher probability of avoiding new lesions), moderate risk (40–60%), and high risk (0–39%) [12].

However, the Cariogram has limitations in assessing a patient’s risk of developing new caries. On the one hand, it is important to consider that this tool is designed to detect late stages of caries progression, whereas current clinical practice emphasizes prevention, early detection, and disease management. On the other, the Cariogram only considers *Streptococcus mutans* species associated with caries development, thus overlooking ecological characteristics of other species relevant to oral health. In addition, the analysis is time-consuming and expensive for the examiner and the patient because it evaluates ten factors, including clinical judgment [21,22]. Furthermore, the number of caries occurrences that may or may not occur in the future is not specified [23,24,25].

Caries Risk Semaphore (CRS) is a web application developed in 2014, as a Project of Docent Innovation, by the Preventive and Community Dentistry unit at the University of Valencia that assesses caries risk by inputting 15 parameters obtained from clinical and radiological examinations, complementary tests (such as bacterial cultures), buffering capacity of saliva, and the patient’s medical and dental history. The program classifies risk into three levels: high (red), medium (yellow), or low (green) based on a diagnostic algorithm developed for it. CRS requires an internet connection but does not need any program installation [26]. Similar to the Cariogram, the limitations of CRS are that it also focuses on *Streptococcus mutans* cultures and may be time-consuming for dental professionals. The application can be found at https://www.uv.es/ieodonto/CRS/index.html (accessed on 30 June 2025).

Early diagnosis of dental caries is crucial for caries management because it is a progressive disease that can permanently damage teeth if left untreated. Early caries detection can prevent the development of more severe cavities, thus avoiding the need for costly and invasive treatments. Utilizing methods such as CAMBRA, the Cariogram, and CRS allows dentists to identify caries at an early stage and apply appropriate treatment, which may include remineralization or fillings. Therefore, early detection using these tools is essential for long-term oral health and preventing more serious dental issues in the future [9,12].

The fundamental basis for managing dental caries is the evaluation of caries risk. Patients’ caries risk can be easily assessed thanks to preventative measures and minimally invasive therapies. As a result, various tools, including CAMBRA, CRS, and the Cariogram, have been developed. Due to differences in risk assessment methodology among these tools, it is essential to study their diagnostic agreement to determine comparability. Thus, the aim of this study is to determine the agreement between different caries risk assessment tools (CAMBRA, CRS, and the Cariogram).

## 2. Materials and Methods

Data were collected from patient questionnaires at the Dental Clinic during Preventive and Community Dentistry II practices, attended by students. Students undergo training among themselves before beginning patient assessments. They perform the assessment numerous times and are corrected by the unit’s professors until they fully understand the criteria (they are assessed theoretically and in practice). Moreover, once they begin examining the patients, the instructors review and correct, if necessary, each examination. This unit is staffed by the faculties experts in the field, who have also participated in many epidemiological studies as explorers and are regarded as the gold standard for calibrating other examiners. The questionnaires collected the factors required for determining caries risk using the three different techniques, including diets habits and oral hygiene practices, among others. Using the three diagnostic tools methods, these data were combined into an Excel spreadsheet to assess each patient’s caries risk. The findings were then contrasted to evaluate their diagnostic agreement. Study participants were patients from 18 to 65 years old, attending recall appointments at the University of Valencia Dental Clinic. Informed consent was provided before initiating examinations. Edentulous patients and incomplete or erroneous data records (because of errors made introducing data of the variables in the database) were excluded from the study. Patients who accepted to participate and signed the informed consent were included in the study.

### 2.1. Ethical Compliance

This study was approved by the Ethics Committee of the University of Valencia, Spain (registration number 2445155). Informed consent was obtained from all subjects involved in the study.

### 2.2. Sample Size Calculation

A sample size of 632 subjects was calculated as suffice to estimate with a 95% confidence level and a precision of +/−4 percentage units, assuming that any of the risk categories reach a 50%. A substitution rate of 5% has been anticipated.

### 2.3. Clinical Examination and Questionnaire

Clinical examinations utilized a dental mirror (number 5) and a PCP 11.5B probe (Hu-Friedy^®^, Chicago, IL, USA). Visual examination employed a bluish-white spectrum light (KaVo^®^ Primus 1058, KaVo^®^, Biberach/Riss, Germany), and air from the equipment’s triple syringe (KaVo^®^, Biberach/Riss, Germany) was used for tooth drying.

Firstly, a questionnaire was completed, collecting information on the patient’s general medical history, oral hygiene habits, intraoral appliance use, social conditions, and daily meal frequency. Then, students recorded the Silness and Löe plaque index by examining the buccal surfaces of teeth 1.6, 1.1, 2.6, 3.6, 3.1, and 4.6. We assigned the following codes: Code 0: No visible plaque; Code 1: Not visible to the naked eye; Code 2: Visible without occupying interproximal spaces; Code 3: Visible and occupying interproximal spaces [27]. The mean value was calculated by summing the points for each surface and dividing by the total number of analyzed surfaces. Additionally, the patient’s caries examination was recorded using the ICDAS II diagnostic criteria, considering caries cutoff point ≥ ICDAS code 4 [28]. The original questionnaire sheets were stored in the patients’ clinical records once the data were collected in an Excel spreadsheet. For the introduction in the spreadsheet, patients were only coded by an identification number.

### 2.4. Radiographic Examination

Two bite-wing radiographs (right and left) were taken for each patient using Kwik Bite bite-wing positioners (Kerr^®^, Detroit, MI, USA) and VistaScan size 2+ radiographic plates (Dürr Dental^®^, Bietigheim-Bissingen, Germany). The patient’s record indicated whether interproximal caries was present or not.

### 2.5. Saliva Collection

The patient’s stimulated saliva was collected for five minutes in a 166cc plastic cup two hours after eating, and the volume of the saliva was quantified. For two minutes, the patient chewed a 1.5 g paraffin tablet, then disposed of the saliva in a spittoon without throwing away the gum. The patient kept chewing the paraffin gum for the following five minutes, collecting the saliva in the plastic cup. Then, a precision digital scale was used to weigh the patient’s saliva container by comparing it to an empty container. To evaluate the volume obtained from stimulated saliva for 5 min, the milliliters have been extrapolated to grams due to its composition like water (total mL/5 min). If the value was below 1 mL/min, the patient had reduced salivary flow; if higher, it was considered normal [29].

We determined saliva buffering capacity with Saliva Check Buffer tests (GC Corporation^®^, Tokyo, Japan) to assess pH, and buffering capacity. We also evaluated *Streptococcus mutans* and *Lactobacillus* sp. colony-forming units (CFUs) using CRT Bacteria cultures (Ivoclar Vivadent^®^, Schaan, Liechtenstein).

To assess buffering capacity and pH, a disposable pipette was used to collect a saliva sample from the patient and it was then deposited on the reactive zones of the Saliva Check Buffer strip (for buffering capacity) and a pH strip, both included in the manufacturer’s kit. After 5 min, we compared the results with the manufacturer’s guide to determine both the saliva pH value and whether the patient had high, medium, or low buffering capacity. These results were also recorded in the patient’s examination sheet.

Finally, with the remaining saliva, a bacterial culture was performed to assess the CFU of *Streptococcus mutans* and *Lactobacillus* Sp. The culture medium from the CRT Bacteria kit was covered with saliva, the reactive tablet was applied to the medium, and it was allowed to incubate for 48 h in an incubator at 37 °C. After this period, the number of CFU for each bacterium should be evaluated by comparison with the manufacturer’s guide.

### 2.6. Statistical Analysis

Once all the data from the different caries risk assessment methods were entered into a Microsoft Excel spreadsheet (Microsoft^®^ Corp., Redmond, WA, USA), they were analyzed using IBM SPSS Statistics v.24 software (IBM^®^, Armonk, NY, USA). Initially, a descriptive analysis was conducted to determine the percentage of the sample classified at each risk level for the three assessment methods. Subsequently, to evaluate agreement, the Kappa statistic was used [30]. The values obtained were categorized as follows: poor agreement for values of 0; slight agreement for values between 0.01 and 0.20; fair agreement between 0.21 and 0.40; moderate agreement between 0.41 and 0.60; substantial agreement between 0.61 and 0.80; and almost perfect agreement between 0.81 and 1.00 [31].

To perform the statistical analysis with the same categories across the different methods, the extreme risk obtained by CAMBRA was integrated into the high-risk category. For the Cariogram, it was established that patients with a probability of avoiding new caries lesions of 61% or more were considered low risk, moderate risk if the probability was between 40% and 60%, and high risk if the probability of avoiding new lesions was between 0% and 39% [12]. To improve the diagnostic agreement between the Cariogram and CAMBRA, optimal cut-off points were calculated to define low/medium/high in the Cariogram based on a receiver operating characteristic (ROC) curve, using CAMBRA as the gold standard.

The CRS already provides its results divided into three categories (low, medium, and high risk), so no recoding was performed.

## 3. Results

For this study, a total of 672 individuals were examined, of which 282 were men (42%) and 390 were women (58%).

In Table 1, Table 2 and Table 3, variables collected for the Cariogram, CRS, and CAMBRA assessments and sample distribution for each variable are presented. With these variables, the three caries risk assessment methods were applied, obtaining the percentage of low, moderate, or high risk for each method.

**Figure 1:**
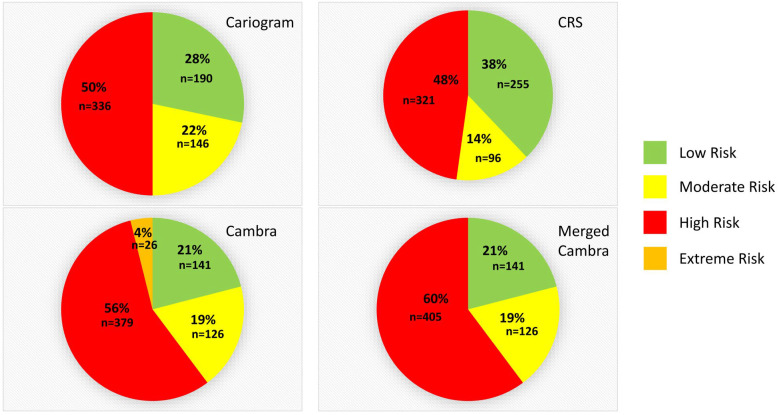
Risk levels (% and sample size) assessed by the Cariogram, Caries Risk Semaphore, CAMBRA, and CAMBRA merging high and extreme risk.

In Figure 1, it can be observed that the Cariogram classified 28% of the patients (190 individuals) as low risk, while CRS classified 38% (255 patients) and CAMBRA classified 21% of the total evaluated individuals (n = 141) in this risk group. For the intermediate risk group, the Cariogram classified 22% of the population (146 individuals), while CRS and CAMBRA classified 14% (96 patients) and 19% (126 patients), respectively. Finally, 50% of the sample (336 patients) were classified as high caries risk according to the Cariogram, 48% (321 individuals) using CRS, and 60% (405 individuals) when merged CAMBRA was employed (high and extreme risk merged). According to this latter evaluation method, it is possible to differentiate between 56% of patients with high risk (n = 379) and the remaining 4% with extreme risk (n = 26).

### Diagnostic Agreement Among Different Models

To assess the diagnostic agreement between the three caries risk assessment models, the aforementioned results were analyzed. New cut-off points for the Cariogram were also calculated using the ROC curve, with CAMBRA serving as the gold standard. These new cutoff points are referred to as ‘Cariogram optimal cutoff point (Cariogram ocp)’ and have the following caries risk ranges: high (25.6% or more), medium (9.6% to 25.5%), and low (0 to 9.5%).

In Table 4, we can observe negative Kappa values in the comparison between CAMBRA vs. Cariogram and CRS vs. Cariogram, with these results improving upon recalculating new cut-off points for the Cariogram. On the other hand, the highest Kappa statistic value is found when comparing CRS vs. CAMBRA, although the value remains low. If we observe the diagnostic agreement between different caries risk assessment methods for each risk level, negative Kappa values are evident in the comparisons between CAMBRA vs. Cariogram and CRS vs. Cariogram, indicating discordance between these systems. However, other comparisons show positive Kappa values, suggesting greater agreement. Notably, the highest Kappa value is found in the comparison CRS vs. CAMBRA.

As shown in Table 5, which compares the parameters included in each caries risk assessment model, there are variables common to all three methods. However, CAMBRA and CRS include the highest number of these variables.

## 4. Discussion

CAMBRA, the Cariogram, and CRS evaluate different parameters, even though some of them are coincident between methods. Except for the *Lactobacillus* sp. count, CAMBRA and CRS include all the variables recorded in the Cariogram, adding the detection of active dentin lesions through clinical and radiographic diagnosis, brushing frequency, and whether the patient uses any type of orthodontic appliance or removable prosthesis. On the other hand, both the Cariogram and CRS include the evaluation of the saliva’s buffering capacity, which is not included in CAMBRA.

In addition to the previously noted differences and similarities, we must additionally account for the subjectivity inherent in the collection and analysis of certain variables. Despite the students who gathered the data being calibrated and supplied with identical materials, we must acknowledge the subjective element and possible inter-examiner discrepancies in caries diagnosis utilizing the ICDAS II code.

Additionally, for radiographic examinations, all students were instructed in the same technique, using a bite-wing parallelizer (Kwik Bite) and a Vistascan^®^ 2+ imaging plate. Despite this standardization, differences in radiograph interpretation may exist among different dentists, leading to potential overestimation or underestimation of caries presence.

Very low Kappa values are observed, indicating that these methods are not directly comparable. The highest Kappa values among the different caries risk assessment methods were found between CRS and CAMBRA, with an unweighted Kappa of 0.36, a linear Kappa of 0.43, and a quadratic Kappa of 0.48. However, even these values are too low to consider these methods as fully comparable. When examining risk stratification (low, medium, and high), we find that the highest Kappa value is also between CRS and CAMBRA with a Kappa for low risk of 0.46, a Kappa for medium risk of 0.14, and a Kappa for high risk of 0.40. These data suggest that the greatest agreement occurs in the low-risk category. However, in the medium-risk group, the very low Kappa indicates substantial disagreement. It is important to note that the challenge is in the classification of patients within this range. For example, CRS offers a direct risk assessment that does not allow for subjective interpretations. In contrast, CAMBRA considers moderate risk when a patient falls between high and extreme risk, resulting in a more subjective evaluation based on clinical reasoning [32]. We can also highlight that the CRS and Cariogram systems are algorithm-based models, while the CAMBRA system relies on clinical reasoning [33,34,35]. It would be reasonable to assume that algorithm-based systems (CRS and Cariogram) should be more comparable and similar to each other. However, the two systems with the highest diagnostic agreement evaluated were CRS and CAMBRA, which both use different methodologies [35]. This factor should be taken into consideration for the design and updating of different caries risk assessment systems and for future research in this area.

In their systematic review, Tellez et al. found variations among caries assessment systems in terms of the definition of risk categories and the type and number of risk or disease factors or indicators [34]. These authors concluded that the Cariogram presented the best combination of sensitivity and specificity for predicting caries in permanent dentition, although this reliability was more limited for preschool-aged niches. This is supported by Su et al., as they conducted a new review in 2021, where two versions of the Cariogram (a complete and a reduced one) were evaluated, concluding that both were reliable for caries prediction [35]. Additionally, they found that the evidence for the predictive capability of CAMBRA is limited, as there are not enough high-quality studies published.

As can be observed, it is not clear which method for assessing caries risk might be the most appropriate. In their narrative review, Twetman et al. stated that no caries risk assessment method has proven to be superior, but they suggest that structured multifactorial models and/or computer-based programs provide the best results [36].

### 4.1. Limitations of the Study

The present study has certain limitations, such as the subjectivity of some of the parameters under study. Additionally, there is an ethical limitation when evaluating the reliability of caries risk assessment methods. If a patient is classified as high risk, it would not be ethical to allow them to continue unchanged, as they could develop caries lesions due to professional oversight [34]. Therefore, it is not possible to determine which instrument would be more reliable, making it challenging to choose the gold standard for clinical application and comparisons with other caries risk assessment methods.

### 4.2. Future Perspectives

To determine which caries assessment method is most suitable for use in our daily clinical practice, further studies on this topic are needed. Since it is ethically unacceptable to allow the patient to continue without modifying the parameters that have classified them as high risk, the monitoring of these patients must be exhaustive. This includes regular revisions and patient interviews at each visit to evaluate the risk factors that are still present. In this way, if new lesions develop, we can assess the factors the patient failed to modify, identify those with the greatest influence, and adjust predictive models accordingly. This will enable a more precise enhancement of caries risk assessment methods.

## 5. Conclusions

A low level of diagnostic agreement has been found between CAMBRA, the Cariogram, and Caries Risk Semaphore. Therefore, we can conclude that the instruments for assessing caries risk are not concordant to each other. This heterogeneity in the study results highlights the need for standardization and uniformity of different evaluation methods to use these procedures reliably in our daily clinical practice.

## Figures and Tables

**Table 1:** Variables collected for the Cariogram assessment and sample distribution.

Variable		Value	%	n
Caries experience	Caries-free and no fillings	0	12.6%	85
	Better than normal	1	19.2%	129
	Normal for age group	2	35.1%	236
	Worse than normal	3	43%	222
Related general diseases	No disease	0	84.7%	569
	Disease/conditions, mild degree	1	11%	74
	Severe degree, long-lasting	2	4.3%	29
Diet, contents	Very low fermentable carbohydrate	0	17.3%	116
	Low fermentable carbohydrate, “non cariogenic” diet	1	46.3%	311
	Moderate fermentable carbohydrate content	2	29.8%	200
	High fermentable carbohydrate intake, inappropriate diet	3	6.7%	45
Diet, frequency	Maximum three meals per day (including snacks)	0	23.1%	155
	Maximum five meals per day	1	51.5%	346
	Maximum seven meals per day	2	10.4%	70
	More than seven meals per day	3	15%	101
Plaque, amount	Extremely good oral hygiene, plaque index, PI < 0.4	0	17.7%	119
	Good oral hygiene, PI = 0.4–1.0	1	41.4%	278
	Less than good oral hygiene, PI = 1.1–2.0	2	32.9%	221
	Poor oral hygiene, PI > 2	3	8.0%	54
*Streptococcus mutans*	Strip mutans class 0	0	34.1%	229
	Strip mutans class 1	1	34.2%	230
	Strip mutans class 2	2	19.2%	129
	Strip mutans class 3	3	12.5%	84
Fluoride program	Receives “maximum” fluoride program	0	17.6%	118
	Additional fluoride measures, measures, infrequently	1	28.3%	190
	Fluoride toothpaste only	2	47.0%	316
	Avoiding fluorides, no fluoride	3	7.1%	48
Saliva secretion-amount	Normal saliva secretion	0	45.5%	306
	Low, 0.9–1.1 mL stimulated saliva/min	1	20.7%	139
	Low, 0.5–0.9 mL saliva/min	2	25.0%	168
	Very low, xerostomia, <0.5 mL saliva/min	3	8.8%	59
Saliva buffer capacity	Adequate	0	56.5%	380
	Reduced	1	28.1%	189
	Low	2	15.3%	103
Clinical judgement	More positive than what the Cariogram shows based on the scores entered	0	10.4%	70
	Normal setting risk according to the other values entered	1	73.4%	493
	Worse than what the Cariogram shows based on the scores entered	2	14.6%	98
	Very high caries risk, examiner is convinced that caries will develop, irrespective of what the Cariogram shows based on the scores entered	3	1.6%	11

n = Sample size.

**Table 2:** Variables collected for the CRS assessment and sample distribution.

Variable	Value	%	n
Active caries in dentin	0	68.9%	463
	1	15.5%	104
	2	6.1%	41
	≥3	9.5%	64
Presence of interproximal caries	0	80.8%	543
	≥1	19.2%	129
Presence of fillings	0	29.9%	201
	1	8.9%	60
	2	9.8%	66
	3	9.8%	66
	4	41.5%	279
Plaque amount	0	18.8%	126
	0.1–0.9	22.9%	154
	1.0–1.9	36.7%	247
	2.0–2.9	16.5%	111
	3	5.1%	34
Prothesis or orthodontics appliance	YES	28.6%	192
	NO	71.4%	480
*Streptococcus mutans* count	HIGH	68.3%	459
	LOW	31.7%	213
Stimulated saliva volume	0	2.1%	14
	<0.5	5.2%	35
	0.5–0.9	18.6%	125
	0.91–1.1	17.4%	117
	>1.1	56.7%	381
Saliva buffer capacity	HIGH	78.7%	529
	LOW	21.3%	143
Cariogenic diet	YES	25.4%	171
	NO	74.6%	501
Number of toothbrushing/day	0	20.1%	135
	1	17.0%	114
	2	36.8%	247
	3	26.6%	176
Adequate fluor exposure	YES	79.5%	534
	NO	20.5%	138
Intake of substances that reduce salivary flow	YES	2.1%	14
	NO	97.9%	658
Radiotherapy treatment	YES	4.9%	33
	NO	95.1%	639
Diseases that reduce salivary flow	YES	5.1%	34
	NO	94.9%	638
Unfavorable social condition	YES	3.0%	20
	NO	97.0%	652

n = Sample size.

**Table 3:** Variables collected for the CAMBRA assessment and sample distribution.

Variable		YES	n	NO	n
Disease indicators (any “Yes” signifies likely “High Risk”)	Cavities (visible or on radiograph) to dentin	32.4%	218	67.6%	454
	Approximal enamel lesions (on radiograph)	17.9%	120	82.1%	552
	Active white-spot lesions on smooth surfaces	13.5%	91	86.5%	581
	Restorations in the past 3 years due to caries	48.5%	326	51.5%	346
Risk factors	High counts of *S. mutans* and *Lactobacillus* sp.	23.2%	156	76.8%	516
	Visible heavy plaque on teeth	61.8%	415	38.2%	257
	Frequent snacking (>3 times daily between meals)	19.6%	132	80.4%	540
	Deep pits and fissures	17.6%	118	82.4%	554
	Recreational drug use	4.2%	28	95.8%	644
	Inadequate saliva flow, measured <1 mL/min stimulated	19.6%	132	80.4%	540
	Saliva-reducing factors (medication/radiation/systemic)	9.5%	64	90.5%	608
	Orthodontic and other appliances with heavy plaque retention potential	7.1%	48	92.9%	624
Protective factors	Lives/works/school fluoridated water	39.6%	266	60.4%	406
	Fluoride toothpaste at least once daily	29.3%	197	70.7%	475
	Fluoride toothpaste at least two times daily	63.8%	429	36.2%	243
	Fluoride mouth rinse (0.05% sodium fluoride) daily	20.4%	137	79.6%	535
	5000-ppm fluoride toothpaste daily	16.4%	110	83.6%	562
	Fluoride varnish in past 6 months	3.3%	22	96.7%	650
	Office fluoride topical in past 6 months	3.1%	21	96.9%	651
	Chlorhexidine prescribed/used 1 week each for past 6 months	8.3%	56	91.7%	616
	Xylitol gum/lozenges 5 times daily past 6 months	3.1%	21	96.9%	651
	Calcium and phosphate paste during past 6 months	3.1%	21	96.9%	651
	Adequate saliva flow (<1 mL/min stimulated)	50.3%	338	49.7%	334

n = Sample size.

**Table 4:** Diagnostic agreement between the three methods assessed by weighted Kappa and its 95% confidence interval.

	Low Risk	Moderate Risk	High Risk	Total
CAMBRA vs. Cariogram	−0.17 (−0.23 to −0.11)	0.04 (−0.04 to 0.12)	−0.18 (−0.25 to 0.11)	−0.23 (−0.30 to −0.16)
CRS vs. Cariogram	−0.18 (−0.25 to −0.11)	0.05 (−0.03 to 0.13)	−0.24 (−0.31 to −0.16)	−0.26 (−0.33 to 0.19)
CRS vs. CAMBRA	0.46 (0.39 to 0.53)	0.14 (0.05 to 0.23)	0.40 (0.34 to 0.47)	0.48 (0.42 to 0.54)
Cariogram ocp vs. CAMBRA	0.25 (0.16 to 0.34)	0.04 (−0.04 to 0.12)	0.23 (0.16 to 0.30)	0.30 (0.22 to 0.37)
Cariogram ocp vs. CRS	0.15 (0.10 to 0.21)	−0.003 (−0.08 to 0.07)	0.25 (0.19 to 0.32)	0.25 (0.19 to 0.31)

CRS: Caries Risk Semaphore. Cariogram ocp: Cariogram for the optimal cutoff point.

**Table 5:** Variables registered for each caries risk assessment tool.

Variables	Cambra	Cariogram	CRS
Radiographic approximal enamel lesions	X		X
Active lesions in dentine	X		X
Orthodontic appliances or removable prosthesis	X		X
Brushing frequency	X		X
Buffer saliva capacity		X	X
*Lactobacillus* sp. count	X	X	
Plaque amount	X	X	X
*Streptococcus mutans* count	X	X	X
Stimulated saliva flow in 5 min	X	X	X
Diet	X	X	X
Fluoride exposure	X	X	X
Saliva-reducing factors	X	X	X

CRS: Caries Risk Semaphore.

## Data Availability

The data presented in this study are available on request from the corresponding author because it contents data from patients still under treatment and or revisions.
